# Comparative evaluation of digital mammography and film mammography: systematic review and meta-analysis

**DOI:** 10.1590/S1516-31802011000400009

**Published:** 2011-05-05

**Authors:** Wagner Iared, David Carlos Shigueoka, Maria Regina Torloni, Fernanda Garozzo Velloni, Sérgio Aron Ajzen, Álvaro Nagib Atallah, Orsine Valente

**Affiliations:** I MD, PhD. Affiliated Researcher at the Brazilian Cochrane Center and radiologist in the Department of Diagnostic Imaging, Universidade Federal de São Paulo (Unifesp), São Paulo, Brazil.; II MD, PhD. Professor in the Department of Diagnostic Imaging, Universidade Federal de São Paulo (Unifesp), São Paulo, Brazil.; III MD, PhD. Gynecologist. Affiliated researcher at the Brazilian Cochrane Center, Universidade Federal de São Paulo (Unifesp), São Paulo, Brazil.; IV MD. Resident in the Department of Diagnostic Imaging, Universidade Federal de São Paulo, Universidade Federal de São Paulo (Unifesp), São Paulo, Brazil.; V MD, PhD. Full Professor of the Department of Diagnostic Imaging, Universidade Federal de São Paulo (Unifesp), São Paulo, Brazil.; VI MD, PhD. Full Professor of the Discipline of Emergency Medicine and Evidence-Based Medicine, Department of Medicine, Universidade Federal de São Paulo (Unifesp), São Paulo, Brazil.; VII MD, PhD. Professor of the Discipline of Emergency Medicine and Evidence-Based Medicine, Department of Medicine, Universidade Federal de São Paulo (Unifesp), São Paulo, Brazil.

**Keywords:** Mammography, Breast neoplasms, Mass screening, Review [Publication type], Meta-analysis [Publication type], Mamografia, Neoplasias da mama, Programas de rastreamento, Revisão [tipo de publicação], Metanálise [tipo de publicação]

## Abstract

**CONTEXT AND OBJECTIVE:**

Mammography is the best method for breast-cancer screening and is capable of reducing mortality rates. Studies that have assessed the clinical impact of mammography have been carried out using film mammography. Digital mammography has been proposed as a substitute for film mammography given the benefits inherent to digital technology. The aim of this study was to compare the performance of digital and film mammography.

**DESIGN:**

Systematic review and meta-analysis.

**METHOD:**

The Medline, Scopus, Embase and Lilacs databases were searched looking for paired studies, cohorts and randomized controlled trials published up to 2009 that compared the performance of digital and film mammography, with regard to cancer detection, recall rates and tumor characteristics. The reference lists of included studies were checked for any relevant citations.

**RESULTS:**

A total of 11 studies involving 190,322 digital and 638,348 film mammography images were included. The cancer detection rates were significantly higher for digital mammography than for film mammography (risk relative, RR = 1.17; 95% confidence interval, CI = 1.06-1.29; I² = 19%). The advantage of digital mammography seemed greatest among patients between 50 and 60 years of age. There were no significant differences between the two methods regarding patient recall rates or the characteristics of the tumors detected.

**CONCLUSION:**

The cancer detection rates using digital mammography are slightly higher than the rates using film mammography. There are no significant differences in recall rates between film and digital mammography. The characteristics of the tumors are similar in patients undergoing the two methods.

## RESUMO

**CONTEXTO E OBJETIVO:**

A mamografia é o melhor método para rastreamento do câncer de mama, capaz de reduzir a mortalidade. Os estudos que avaliam seu impacto clínico foram realizados com mamografia em filme. A mamografia digital é proposta para substituir a mamografia em filme com benefícios inerentes à tecnologia digital. O objetivo do estudo foi comparar o desempenho da mamografia digital com a mamografia em filme.

**TIPO DE ESTUDO:**

Revisão sistemática e metanálise.

**MÉTODO:**

Foram pesquisadas as bases Medline, Scopus, Embase e Lilacs, buscando-se por estudos pareados, coortes e ensaios clínicos randomizados comparando a mamografia digital e a mamografia em filme, quanto à taxa de detecção de câncer, de reconvocação e características dos tumores, publicados até 2009. As referências dos estudos incluídos foram verificadas em busca de citações relevantes.

**RESULTADOS:**

Foi incluído um total de 11 estudos, somando 190.322 mamografias digitais e 638.348 em filme. A taxa de detecção do câncer pela mamografia digital foi significantemente maior (risco relativo, RR: 1,17 [95% intervalo de confiança, IC = 1,06-1,29 I² = 19%]) do que pela mamografia em filme. A vantagem da mamografia digital parece maior em pacientes entre 50 e 60 anos. Não houve diferenças significantes nas taxas de reconvocação de pacientes e nas características dos tumores encontrados.

**CONCLUSÃO:**

A mamografia digital apresenta taxa de detecção de câncer pouco maior que a mamografia em filme. Não há diferenças significantes nas taxas de reconvocação entre a mamografia digital e a em filme. As características dos tumores são semelhantes em pacientes em ambos os métodos.

## INTRODUCTION

Breast cancer is the second most frequent malignant neoplasm among the female population.^1^ In Brazil, it has been estimated that there are 50.71 cases for every 100,000 women, second only to non-melanoma skin neoplasms. The specific mortality rate is 11.4 per 100,000 women, which means that, each year, more than 10,000 women die from this disease.^2^ There is evidence in the medical literature that suggests that periodic mammography screening is the most effective method for early diagnosis of breast cancer, with significant reductions in the specific mortality rates caused by this disease. The evidence indicates that this screening method is especially beneficial for women aged 50 and over, although there are studies showing that this method also provides significant benefits for women between 40 and 49 years of age.^3–6^

One recent historic landmark in mammography was the United States Food and Drug Administration's (FDA's) approval of the first full-field digital equipment for breast-cancer screening, in January 2000.^7^ Initially, digital technology was merely used to orient mammography-guided intervention procedures, in which detectors allowed physicians to obtain images from a small field of view. Full-field digital mammography is intended to replace film mammography in screening and diagnosing breast cancer. Digital technology brings with it a series of implicit benefits, which include storage of images in digital databases, without an ensuing loss of quality, and the ability to transmit images over long distances. One of the factors that most compromises film mammography is the fact that the images need to be developed and fixed chemically, and the image rejection rate due to processing errors can surpass 20%.^8^ The need to repeat these examinations increases costs and exposes patients to higher doses of ionizing radiation. Digital mammography does away with the chemical processing of images and, by enabling correction of brightness and contrast, can potentially reduce the rate of image rejection due to technical errors.

Other benefits inherent to digital technology are: the ability to amplify images on a monitor without the need to subject the patient to further X-ray exposure for magnified imaging; the ability to subsequently manipulate image contrast and brightness, and to use filters (software that selects or excludes certain gray-scale tones); and the ability to make computer-aided diagnoses using specific software that recognizes image patterns in lesions. However, all studies that demonstrate the effectiveness of mammography for reducing breast-cancer mortality have been based on traditional, film mammography.^3^ A large proportion of the studies that assess digital mammography have focused on technical data, like spatial resolution, contrast details and the calculable efficiency of the detector.^9–11^ To date, none of the studies that have compared the performance of digital mammography and film mammography have assessed the impact of digital mammography specifically in terms of mortality. This type of study may take many years or even decades. The studies that are currently available merely assess intermediate or substitute endpoints, like cancer detection rates, patient recall rates and the clinical characteristics of the tumors detected.^12–14^

## OBJECTIVES

The objective of this systematic review of the scientific literature was to compare the performance of digital mammography and film mammography in terms of cancer detection rates, patient recall rates and characteristics of the tumors detected.

## METHODS

### Type of study

Systematic review of the scientific literature and meta-analysis.

### Types of studies included

Paired studies, cohort studies and clinical trials comparing film mammography and digital mammography that were published up to September 2009.

### Types of participants

Women age 40 or over who were enrolled in breast cancer screening programs or who complained of specific ailments, and for whom mammography was recommended.

### Types of endpoints

The following endpoints were assessed:

- Cancer detection rate;
- Patient recall rate;
- Characteristics of the tumors detected.

The cancer detection rate is the ratio between the number of cancer cases confirmed through biopsy (histopathological analysis) and the number of cases detected by each method, in all patients screened.

The recall rate is the proportion of the patients with images that are sufficiently suspect to require the patients to be called in again for further screening, additional propaedeutic investigation and possible biopsy (histopathological analysis).

In order to compare the characteristics of the tumors found, we evaluated the ratio of invasive tumors to *in situ* tumors found using each method.

### Subgroup analysis

The following subgroup analyses were conducted:

- Cancer detection rate in patients 60 years old or younger;
- Cancer detection rate in patients older than 60 years of age;
- Cancer detection rate in patients between 45 and 49 years old.

### Inclusion criteria

All studies that compared film and digital mammography in similar populations in terms of the aforementioned endpoints were included in this study.

### Exclusion criteria

Studies presenting any of the characteristics listed below were excluded:

- Studies that assessed the detection rate for just one of the methods.
- Studies from which it was impossible to extract cancer detection data and recall data relating to one of the mammography methods.
- Studies in which cases of previously reported cancer(s) were included in the study sample.
- Studies that assessed merely one of the technical parameters.
- Studies that did not include an abstract in English, Portuguese or Spanish in the databases consulted.
- Studies that examined subpopulations of larger studies previously included in this review.

### Location

This meta-analysis was conducted at the Brazilian Cochrane Center at the Universidade Federal de São Paulo — Escola Paulista de Medicina (Unifesp-EPM), within the Emergency Medicine and Evidence-Based Medicine Program of the Department of Medicine.

### Search strategy

The search strategy involved searching four electronic databases (Medline via PubMed, Embase, Lilacs and Scopus) for articles on the topics of digital and film mammography that had been published up to September 2009. The bibliographic references of the studies included were checked in order to search for additional potentially relevant citations. The search strategy was sensitive to text and abstract wording, was unfiltered and used the following strategies:

#### Databases: Medline via PubMed, Embase and Scopus

#1: MAMMOGRAPHY

#2: DIGITAL

#3: #1 AND #2

#### Lilacs Database

#1: MAMMOGRAPHY

#2: MAMOGRAFÍA

#3: MAMOGRAFIA

#4: #1 OR #2 OR #3

#5: DIGITAL

#6: #4 AND #5

### Study locations

Two reviewers (MRT and WI or FV and WI) independently assessed the titles and summaries of all the resulting citations identified in the electronic search. Studies that potentially met the inclusion criteria were then read in full. Divergent opinions were resolved by reaching a consensus. Studies for which no consensus was reached were considered potentially eligible.

### Data extraction

All studies with inclusion potential were separated for a full reading, critical assessment and data extraction, and this was done independently by two reviewers (DCS and WI).

A specific form was created for extracting the data from each study, and the following data were gathered: general information on each study (author and publication year), type of study, type of patients, study location, total number of cases, number of recall cases, total number of cases with a cancer diagnosis, positive predictive value and total numbers of *in situ* carcinomas and invasive carcinomas. Where included, these same data were stratified according to age group.

The data extracted by each researcher were inserted into individual spreadsheets that were subsequently compared. Divergent opinions were resolved by reaching a consensus.

### Statistical analysis

We expressed the differences in cancer detection rates, patient recall rates and *in situ* and invasive cancer rates as relative risk (RR) rates using the statistical random-effects model for dichotomous data.

Risk ratios and the respective 95% confidence intervals were calculated. The data were compared using forest plots.

Review Manager 5.0.20, which is a computer software freely distributed by the Cochrane Collaboration, was used for the calculations and to generate the plots.

The heterogeneity of the estimated effects among the studies included was analyzed using the heterogeneity or inconsistency test (I²). I² values less than 30% were considered to be indicators of low heterogeneity; values between 30-50% were considered to be indicators of moderate heterogeneity; and values above 50% were considered to be indicators of high heterogeneity.

## RESULTS

### A. Studies included

The electronic search identified 1,644 bibliographic citations: 1,203 references in Medline, 214 in Embase, 198 in Scopus and 29 in Lilacs. After reading the titles and abstracts, and after eliminating duplicates, 26 articles were chosen for a full reading and critical assessment. Of these, 15 were excluded because they did not meet the inclusion criteria or because they met the exclusion criteria. Table 1^12,15–28^ summarizes the reasons for excluding these 15 studies.

**Table 1.** Main reasons for excluding studies from the systematic review^12,15–28^

Author	Reason for exclusion
Cole et al.^15^	Assessment of cases with previously known cancer diagnoses
Hendrick et al.^28^	Retrospective study using data from Pisano's 2005 Digital Mammographic Imaging Screening Trial (DMIST)^14^ study
Venta et al.^17^	Did not assess clinical data of interest for this systematic review
Lewin et al.^18^	Study conducted with part of the population of the complete study,^30^ which was published the following year and has been included in the systematic review
Nishikawa et al.^19^	Retrospective study using data from Pisano et al.^14^ DMIST study
Onishi et al.^20^	Study of population with prior surgical recommendation
Pisano et al.^16^	Retrospective study using data from Pisano et al.^14^ DMIST study
Ranganathan et al.^21^	Did not report data for calculating cancer detection rates and recall rates
Seo et al.^22^	Assessment using previously known mammary lesions
Skaane et al.^23^	Study that assessed endpoints other than those listed in the objective of this review, using data from the Skaane et al. ^31^ and Skaane et al.^29^ Oslo I^12^ and Oslo II^13^ studies
Skaane et al.^12^	First published paper from the study data included in the Skaane et al.^31^ Oslo I^12^ study
Skaane et al.^24^	Study that assessed endpoints other than the objective of this review, using data from the Skaane et al.^31^ and Skaane et al.^29^ Oslo I^12^ and Oslo II^13^ studies
Tosteson et al.^25^	Study that assessed endpoints other than those listed in the objective of this review (cost effectiveness)
Yamada et al.^26^	Study that assesses endpoints other than those listed in the objective of this review (technical parameters)
Yamada et al.^27^	Known cancer cases included

**Figure 1:**
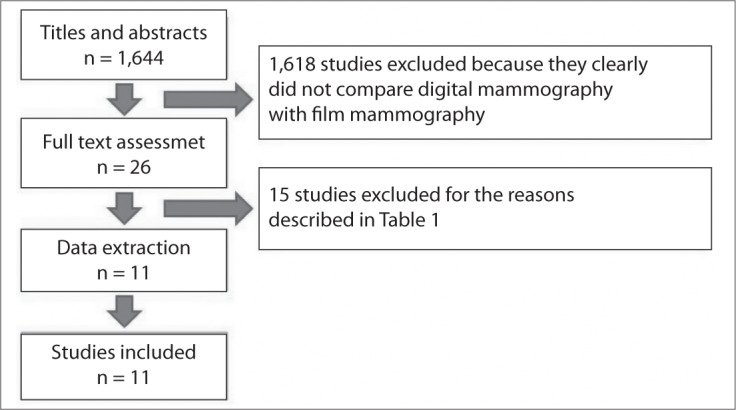
Flowchart for the process of study inclusion in the review.

Upon completion of the search strategy and recovery, 11 studies were ultimately included in the systematic review (Figure 1).

Among the studies included, we found one randomized controlled trial (RCT),^29^ three paired studies (prospective studies in which all the patients included underwent both types of mammography)^14,30,31^ and seven cohort studies.^32–38^

The allocation concealment in the only RCT included was not clearly described in the published paper.^29^

The cohort studies included a total of 130,199 digital mammography images and 568,184 film mammography images. From the digital images, 801 cancers were detected; and from the film images, 3,341 cancers were detected.^32–38^

The RCT included 6,944 digital mammography images and 16,985 film mammography images. From these, 41 and 64 cases of cancer were detected in the groups using digital and film mammography respectively.^29^

The paired studies included a total of 53,179 digital mammography images and film mammography images. Digital mammography was able to detect 182 cases of cancer, while film mammography was able to detect 194 cases.^14,30,31^

Table 2^14,23,29,30,32–38^ summarizes the main characteristics of the studies included.

**Table 2.** Main characteristics of the studies included in the systematic review^14,23,29,30,32–38^

	Study design	Participants	Comparison	Endpoints assessed	Study date	Study location
Del Turco et al.^32^	Cohort study, retrospective	Women aged 50 to 69 in a breast cancer screening program	Digital versus film mammography	Cancer detection rate, patient recall rate and tumor characteristics. Assessment of subgroups by age group: 50-59 years; 60-69 years	January 2004 to October 2005	Florence, Italy
Hambly et al.^38^	Cohort study, retrospective	Women aged 50 to 64 years old invited to participate in a screening program	Digital versus film mammography	Digital versus film mammography	January 2005 to December 2007	Ireland
Heddson et al.^33^	Cohort study, retrospective	Women in breast cancer screening program. Maximum age of 74 years, variable minimum age	Digital versus film mammography	Cancer diagnosis rate, patient recall rate and positive predictive value	January 2000 to February 2005	Sweden

Lewin et al.^30^	Paired study, prospective	Women older than 40 years of age who came in for screening at either of the two centers. Symptomatic patients excluded.	Digital versus film mammography	Cancer detection rate and patient recall rate	2000 and 2001 (estimated)	United States
Pisano et al.^14^	Paired study, prospective	49,528 patients who came in for screening at the participating institutions	Digital versus film mammography	Sensitivity, specificity, positive predictive value and negative predictive value of the screening methods	October 2001 to November 2003	Thirty-three participating locations in United States and Canada
Sala et al.^37^	Cohort study, retrospective	Women aged 50 to 69 years in a screening program	Digital versus film mammography	Cancer detection rate and patient recall rate	February 2002 to January 2007	Barcelona, Spain
Skaane et al.^23^	Paired study, prospective	Women aged 50 to 69 years in a screening program	Digital versus film mammography	Cancer detection rate and patient recall rate	January 2000 to June 2000	Oslo, Norway
Skaane et al.^29^	Randomized trial, prospective	Women aged 45 to 69 years in a screening program	Digital versus film mammography	Recall rate, cancer detection rate, positive predictive value, sensitivity, specificity, tumor characteristics and discordant interpretations	November 2000 to December 2001	Oslo, Norway
Vernacchia et al.^36^	Cohort study, retrospective	Women aged 40 years or older in a screening program	Digital versus film mammography	Cancer detection rate and patient recall rate	July 2004 to August 2008	San Luis Obispo, California, United States
Vigeland et al.^34^	Cohort study, retrospective	Women aged 50 to 69 years in a screening program	Digital versus film mammography	Cancer detection rate, patient recall rate and positive predictive value	February 2004 to December 2005	Vestfold, Norway
Vinnicombe et al.^35^	Cohort study, retrospective	Women aged 50 years or older in a screening program	Digital versus film mammography	Cancer detection rate, patient recall rate and positive predictive value	January 2005 to June 2007	London, United Kingdom

### B. Effectiveness of digital mammography compared with film mammography for detecting breast cancer

The results showed homogeneity in terms of the cancer detection rate. The cancer detection rate was significantly higher among patients who underwent digital mammography.

Based on the combination of data from the 11 studies included in this systematic review, the average relative-risk estimate for cancer detection among patients who underwent digital mammography was 1.17 (95% confidence interval, CI = 1.06-1.29; I² = 19%), in relation to film mammography.

The combined RR, considering merely the seven cohort studies, was 1.21 (95% CI = 1.11-1.32; I² = 0%), and the RR for the RCT was 1.57 (95% CI = 1.06-2.32).

On the other hand, taking into consideration only the paired studies, there was no significant difference in cancer detection rates between the two methods: average RR of 0.94 (95% CI = 0.77-1.15; I² = 0%).

**Figure 2:**
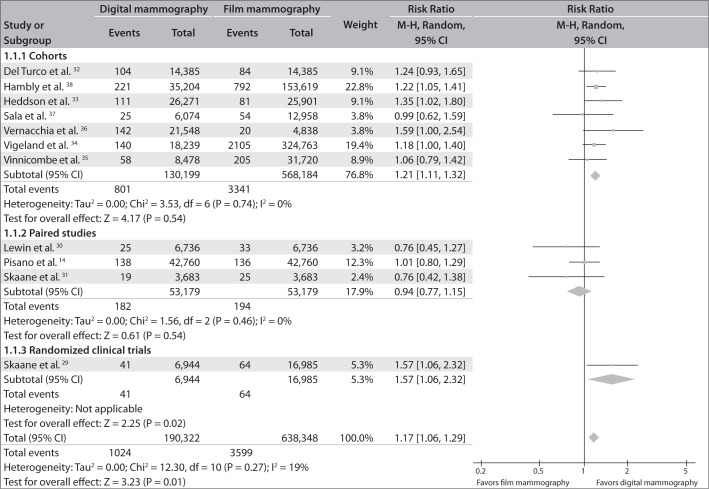
Breast cancer detection rates from digital versus film mammography.

Figure 2 shows the forest plot with the same information.

### C. Recall rate for digital mammography compared with film mammography

There was great heterogeneity among the studies with regard to the patient recall rate (I² = 96%), even when they were analyzed according to study design (for cohort studies, I² = 95%; for paired studies, I² = 93%).

The meta-analysis did not identify any significant difference between the two methods with regard to the patient recall rate: RR = 1.07; 95% CI = 0.94-1.22; I² = 96%). However, the RCT revealed a significant difference, with higher recall rates among patients who underwent digital mammography (RR = 1.69; 95% CI = 1.46-1.96).

**Figure 3:**
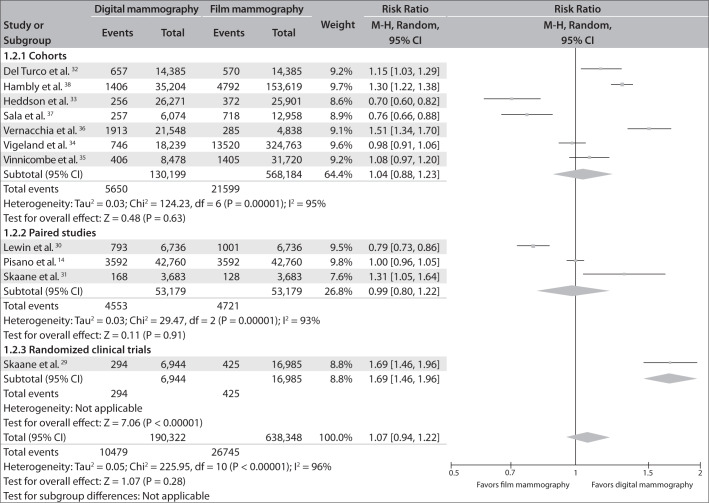
Patient recall rates from digital versus film mammography.

Figure 3 shows the same information in a forest plot.

### D. Characteristics of the tumors found using digital mammography, compared with film mammography

The characteristics of the tumors were seen to be similar from the two methods, in studies that provided this information. The relative risk of the proportion of invasive tumors in relation to the total number of tumors was 0.97 (95% CI = 0.91-1.04; I ² = 42%). Likewise, the relative risk of the proportion of *in situ* carcinomas was 1.14 (95% CI = 0.88-1.47, I ² = 48%).

**Figure 4:**
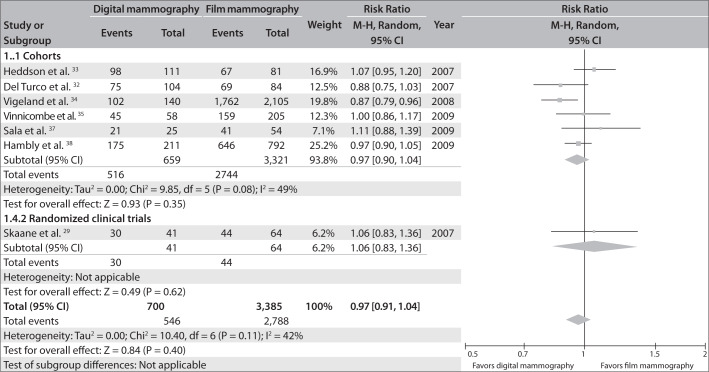
Invasive carcinoma detection rates from digital versus film mammography

**Figure 5:**
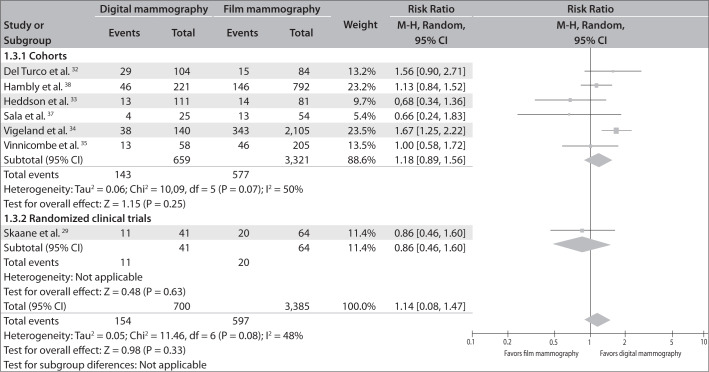
*In situ* carcinoma detection rates from digital versus film mammography.

Figures 4 and 5 present the same information in the form of a forest plot graph.

### E. Breast cancer detection rates in different age groups, comparing digital and film mammography

**Figure 6:**
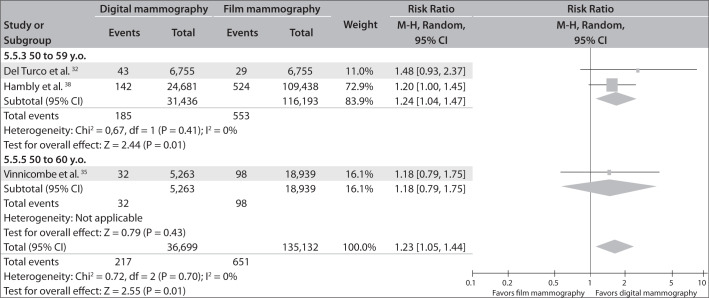
Breast cancer detection rates comparing digital mammography with film mammography in subgroups of patients between 50 and 60 years old.

**Figure 7:**
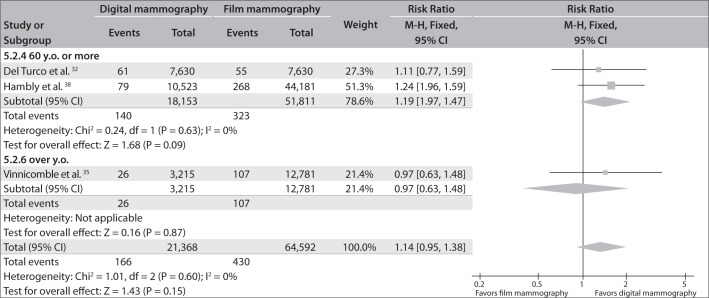
Breast cancer detection rates comparing digital mammography with film mammography in subgroups of patients aged 60 or over.

Subgroup analysis according to age group, in the cohort studies that included this information,^32,35,38^ revealed that digital mammography was better than film mammography for detecting tumors in patients between 50 and 60 years of age (RR = 1.23; 95% CI = 1.05-1.44; I² = 0%). No significant differences were identified in groups older than 60 years of age (RR = 1.14; 95% CI = 0.95-1.38; I² = 0%) (Figure 6 and Figure 7).

**Figure 8:**
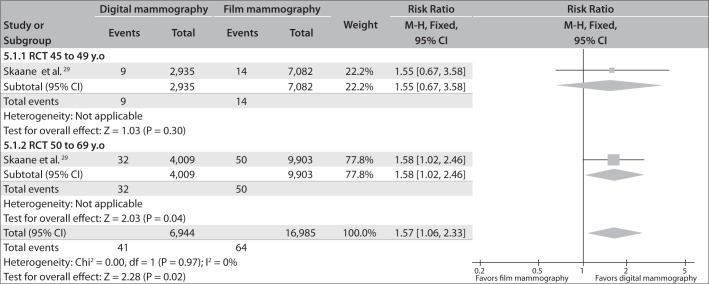
Breast cancer detection rates comparing digital mammography with film mammography in subgroups of patients between 45 and 49 years old and patients between 50 and 69 years old.

The RCT assessed patients between the ages of 45 and 49 years as well as patients older than 50 years. This trial found that digital mammography was more effective only in the older age group: RR = 1.58; 95% CI = 1.02 - 2.46; versus RR = 1.55; 95% CI = 0.67-3.58, respectively (Figure 8).

Moreover, despite the data available from the RCT did not allow its inclusion in the meta-analysis, Pisano, in the DMIST study^14^, reported that digital mammography showed greater accuracy for perimenopausal and premenopausal patients and in patients younger than 50 years with higher mammary density.

## DISCUSSION

While mammography is a diagnostic test, studies that address the spectrum of patients of interest in clinical practice (women in breast cancer screening programs) are technically and ethically limited in terms of traditional methodology. The main limitation is the lack of a reference standard (a “gold standard”) that is acceptable for negative tests. While a biopsy can be taken of the suspect lesion to confirm or reject the presence of cancer, if mammography images on the entire mass of both breasts come up negative, such patients cannot be subjected to histopathological study. Thus, studies lack information on the number of false negative cases. Without this information, the sensitivity, specificity and negative predictive value cannot be reported. Even the positive tests confirmed by biopsy do not assess the contralateral breast or other regions of the breast with the suspect lesion. Thus, without being able to create a 2 x 2 table, there are serious limitations with regard to obtaining data regarding sensitivity, specificity and negative predictive value. On the other hand, positive predictive values can be obtained. Nonetheless, the criteria for determining that a test is positive or suspect are extremely variable and do not follow specific standards in different clinics, as seen by the extreme heterogeneity of recall rates found in the studies included in this meta-analysis.

The most appropriate means of assessing the effectiveness of mammography for screening for breast cancer is to interpret it like an intervention, such that the primary endpoint is the reduction in mortality rates. Since digital mammography is a relatively new method, it may take decades until there are sufficient data to compare this endpoint with findings from film mammography. The primary focus of the present systematic review was a surrogate outcome, i.e. the cancer detection rate.

Following the FDA's approval of digital mammography in January 2000,^7^ several studies comparing this method and film mammography were published. More recently, two systematic reviews on the matter were published. One, in 2007, included data from oral presentations given in congresses, in addition to published studies;^39^ and the other, in 2009, also included the results from a cohort study.^35^ Before the present review had been completed, other studies not included in earlier reviews were published, including a large cohort study with more than 188,000 women, of whom 35,000 had undergone digital mammography. Thus, updating the reviews is justified in the name of strengthening the degree of evidence available for subsequent decision-making.

It can be seen that, in older studies published up to 2005, there were no significant differences in cancer detection rate between the two types of mammography. However, the majority of the studies published from 2007 onwards have reported a significantly higher detection rate from digital mammography, compared with film mammography. One hypothesis for explaining this difference may relate to small technological advances in the equipment and to the learning curve for radiologists who use digital technology.

The subgroup assessment according to age groups showed that the superiority of digital mammography in terms of the cancer detection rate was more evident in the 50-60 year old group. This difference decreased and became insignificant in populations aged 60 years or over. The assessment of the population between 45 and 50 years of age in the RCT also did not demonstrate any significant difference. One possible explanation for this is that mammary density is higher in populations of intermediate age than in women aged 60 or older, and that digital resources, like the use of filters and contrast manipulation, probably influence cancer detection. In women with greater lipid replacement in the mammary parenchyma, which is to be expected in older age groups, these image manipulation resources do not provide additional value. On the other hand, in younger populations, the density of the mammary parenchyma is probably too high for both types of mammography, thus leading to similar cancer detection rates.

There was significant heterogeneity in the study results in terms of patient recall rates, which can be defined as the need for additional investigations to define the diagnosis of cancer. We believe that particular differences in the criteria used to recall patients, which were not standardized, were responsible for this difference in rates.

With regard to the advantages associated with digital technology, the process of replacing film mammography with digital mammography is slow. This is partially explained by the still-high initial costs associated with digital equipment and partially by a lack of trust in the capacity of new technology to detect breast cancer with the same accuracy as the already-revered method of film mammography.

The DMIST study contained an estimate that the cost of digital mammography systems was 1.5 to 4 times the cost of film mammography systems.^14^ However, some authors believe that the replacement of film systems with digital systems is inevitable and that the path to its acceptance is one from which there is no turning back.^40^ Moreover, it is well-known that the natural tendency is for the price of new technology to drop over time.

The results from this systematic review showed that there is a small difference in cancer detection rates in favor of digital mammography. However, the most important finding was that there is no evidence to support any claim that digital mammography is inferior to film mammography in terms of cancer detection rates.

The possibility of long-distance transmission, which enables assessment by specialists located thousands of miles away from the examination center, is an important element of the digital system to consider in developing countries or in places with a reduced number of radiologists experienced in mammography.

## CONCLUSION

Digital mammography presents cancer detection rates that are slightly higher than the rates shown by film mammography. There are no significant differences in patient recall rates between digital and film mammography. The characteristics of the tumors found are similar in patients who undergo digital and film mammography.
